# Percutaneous Cannulated Screw Fixation vs. Plating With Minimally Invasive Longitudinal Approach After Closed Reduction for Intra-Articular Tongue-Type Calcaneal Fractures: A Retrospective Cohort Study

**DOI:** 10.3389/fsurg.2022.854210

**Published:** 2022-04-04

**Authors:** Yuan Cao, Xiangyu Xu, Yan Guo, Zengzhen Cui, Yang Zhao, Shan Gao, Yun Tian, Yang Lv, Fang Zhou

**Affiliations:** ^1^Department of Orthopaedics, Peking University Third Hospital, Beijing, China; ^2^Engineering Research Center of Bone and Joint Precision Medicine, Beijing, China; ^3^School of Basic Medical Sciences, Peking University Health Science Center, Beijing, China

**Keywords:** calcaneus, tongue-type fracture, joint depression type, closed reduction, percutaneous cannulated screw fixation, minimally invasive longitudinal approach

## Abstract

**Objective:**

Displaced intra-articular tongue-type fractures are often treated with surgical interventions, and there is a lack of consensus regarding the surgical approach. This retrospective cohort study aimed to compare percutaneous cannulated screw (PCS) fixation and plating with a minimally invasive longitudinal approach (MILA) after closed reduction for the treatment of tongue-type calcaneal fractures.

**Materials and Methods:**

A total of 77 patients with intra-articular tongue-type calcaneal fractures between September 2015 and July 2019 were included in this study. They were randomly allocated into two groups: PCS fixation (*n* = 32) and MILA (*n* = 45). The outcome measures included demographic variables, operation time (OT), preoperative time (POT), hospital stay time (HST), blood loss, visual analog scale (VAS) scores, American Orthopaedic Foot and Ankle Society (AOFAS) hindfoot scores, postoperative complications, and imaging parameters. The patients were clinically examined at 1, 3, 6, and 12 months, with a final follow-up period of 27 months.

**Results:**

No significant differences were observed during the follow-up in calcaneal length, height, Gissane's and Böhler's angles, VAS scores, AOFAS hindfoot scores, or complication rates between the two groups. However, the postoperative VAS scores in the PCS group were significantly lower than those in the MILA group (*p* < 0.05). Furthermore, the OT, POT, and HST in the PCS group were significantly shorter than those in the MILA group (*p* < 0.05). Blood loss was lower in the PCS group than those in the other group (*p* = 0.044). However, postoperative calcaneal widening was significantly smaller in the MILA group than that in the PCS group (*p* < 0.001).

**Conclusions:**

After closed reduction for the treatment of tongue-type calcaneal fractures, PCS fixation was superior to MILA in terms of OT, POT, HST, blood loss, pain, and degree of comfort. Meanwhile, MILA has the advantage of restoring the calcaneal width. Under the same rehabilitation program, the two approaches showed similar abilities in maintaining the closed reduction.

## Introduction

Calcaneal fractures are a common type of fracture, accounting for >60% of fractures in tarsal bones (1). Calcaneal fractures are often caused by high-impact trauma, such as a fall from a height or traffic accident. Approximately 75% of calcaneal fractures are of the intra-articular type. Essex–Lopresti divided displaced intra-articular calcaneal fractures into the tongue and joint depression types (2). Tongue-type patterns extend into the subtalar joint, and the secondary fracture line exits posteriorly through the calcaneal tuberosity.

The displacement of intra-articular fractures usually requires surgical therapy to establish anatomical reduction and subtalar joint alignment. However, there is a lack of consensus regarding the surgical methods. The standard surgical approach is open reduction and internal fixation via an extensile L-shaped incision (3); however, complications, such as wound dehiscence and deep infection, which may result in delayed wound healing, frequently occur (4). To decrease the rate of complications, diverse minimally invasive techniques have been proposed, including arthroscopically assisted fixation, external fixation, percutaneous fixation, and various minimal incision techniques (5–10). Closed reduction is an appropriate choice for intra-articular tongue-type calcaneal fractures because the fracture block is large and can be easily reduced using leveraging technology (1).

The sinus tarsi approach is one of the most widely used minimally invasive techniques. Zhang et al. (9) reported that for the treatment of Sanders types II and III displaced intra-articular fractures of the calcaneus, the minimally invasive longitudinal approach (MILA) resulted in a lower complication rate and shorter operative time than the sinus tarsi surgical approach. Feng et al. (10) reported that percutaneous cannulated screw (PCS) fixation is superior to the sinus tarsi approach with respect to the subtalar joint activity. However, few studies have investigated the difference between PCS fixation and plating using the MILA. On one hand, the incision of plating is large, which may also damage the nerves and vessels, and the plates need to be removed after rehabilitation. However, PCS fixation has none of these disadvantages, although it is not as strong as plating and may cause fracture displacement. The primary goal of this study was to compare PCS fixation with plating using the MILA after closed reduction for intra-articular tongue-type calcaneal fractures.

## Materials and Methods

This retrospective cohort study was conducted from September 2015 to July 2019. In total, 86 patients who underwent the PCS fixation or MILA after closed reduction at Peking University Third Hospital were included in this study. The study was approved by the Medical Ethics Committee of the Peking University Third Hospital, and written informed consent was obtained from all included patients prior to surgery. The study inclusion criteria were as follows: (1) age of 18–60 years; (2) presence of closed fractures; and (3) presence of intra-articular tongue-type calcaneal fractures. Exclusion criteria were as follows: presence of (1) joint depression-type fractures, (2) open or pathological fractures, or (3) bilateral calcaneal fractures or a history of (4) previous ankle surgery, ankle, or talar fractures, (5) severe osteoporosis, or (6) diabetes mellitus. Of the 86 patients, nine were lost to follow-up because they could not be contacted or did not finish the follow-up protocol; 77 patients (89.5%) were included in the final analysis.

Two surgical methods were randomly performed on these patients according to the surgeon's preference. Therefore, the patients were retrospectively divided into two groups: the PCS group (*n* = 32), who underwent PCS fixation after closed reduction, and the MILA group (*n* = 45), who underwent plating using the MILA after closed reduction. All patient data were evaluated immediately after surgery although except during the 1-, 3-, 6-, and 12-month follow-up visits.

### Preoperative Management

Preoperative calcaneal radiographs, computed tomography (CT) scans, and three-dimensional reconstruction of the injured foot were performed to evaluate the fractures. The MILA group underwent surgery after the hindfoot swelling subsided; however, the PCS group could be operated on directly. Under either epidural or spinal anesthesia, both groups were placed in the lateral decubitus position using tourniquets. All patients were operated upon by the same team of surgeons.

### Surgical Technique

**Figure 1:**
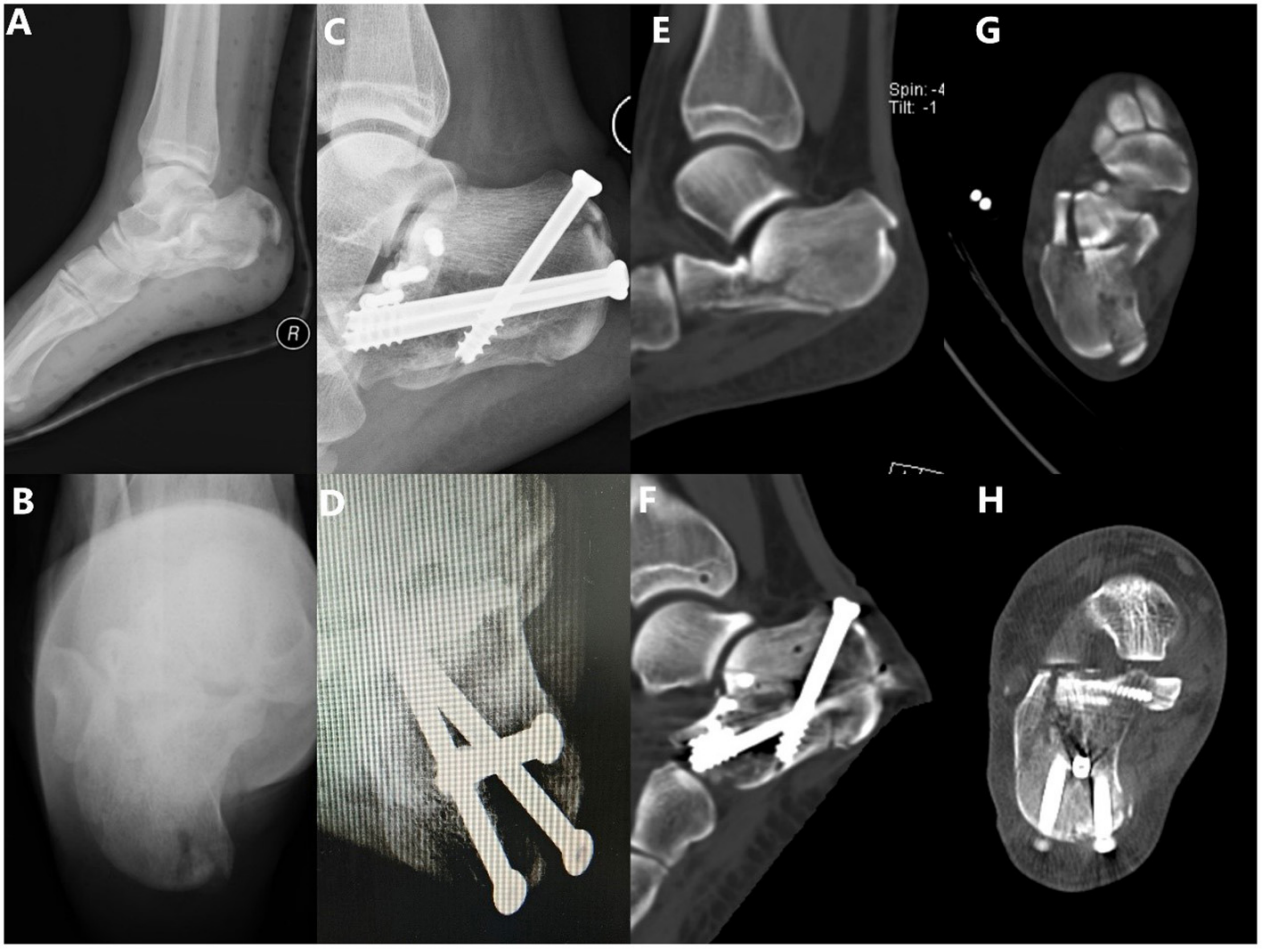
A 19-year-old female patient who fell from a height, resulting in a tongue-type intra-articular fracture of the calcaneum. **(A)** Preoperative lateral X-ray and **(B)** preoperative axial X-ray presenting an intra-articular tongue-type calcaneal fracture with subtalar articular surface collapse, loss of Böhler's angle and Gissane's angle, significantly decreased calcaneal height/length, and significantly increased calcaneal width. **(C)** Postoperative lateral X-ray showing the reduction of calcaneal length, height, Böhler's angle, and Gissane's angle. **(D)** Postoperative axial X-ray showing the dissatisfactory restoration of the calcaneal varus. **(E,G)** The preoperative computed tomography (CT) scan shows a Sanders type II fracture. **(F,H)** Postoperative CT scan shows the reduction of the articular surface was satisfactory, but the restoration of the calcaneal width was dissatisfactory.

**Figure 2:**
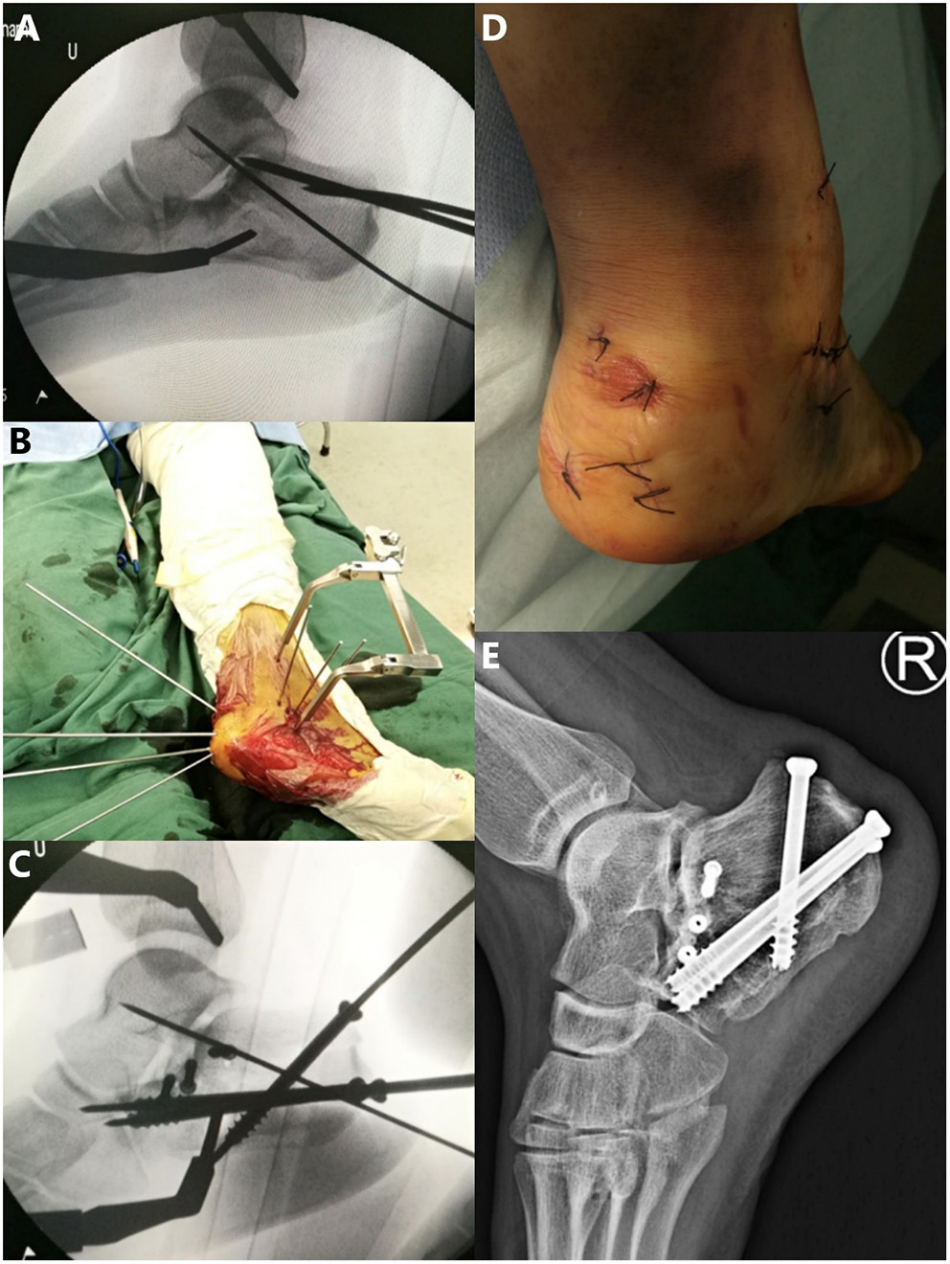
**(A)** In the operation on the foot in Figure 1, the Kirschner wire (2.0 mm) was inserted into the talus through the fracture line and the posterior face to maintain the reduction after the fracture was reduced by percutaneous leverage. **(B,C)** Guide pins were replaced percutaneously by appropriately sized cannulated screws. **(D)** Postoperative incision. **(E)** Lateral X-ray 12 months after the surgery.

First, in the PCS group, two Steinmann pins (3.0 mm) were inserted through the calcaneal tuberosity into the fractured tongue fragment at a 60° angle to the plantar surface on both sides of the Achilles tendon. The tip of the Steinmann pins was stopped inside the fracture line of the posterior facet of the subtalar joint. The midfoot was bent toward the plantar surface with one hand, and the pin was held with the other hand to restore the posterior facet. Meanwhile, the assistant squeezed the calcaneus to reconstruct its width and reduce varus of the calcaneus. After the closed reduction was confirmed under C-arm guidance, two Kirschner wires (2.0 mm) were inserted parallel to the talus through the fracture line and posterior face to maintain the reduction. Under C-arm fluoroscopy guidance, three guide pins were introduced from the lateral calcaneus to the medial calcaneus, and two other guide pins were inserted through the calcaneal tuberosity. Finally, the guide pins were percutaneously replaced with appropriately sized cannulated screws, and the wounds were closed (Figures 1, 2).

**Figure 3:**
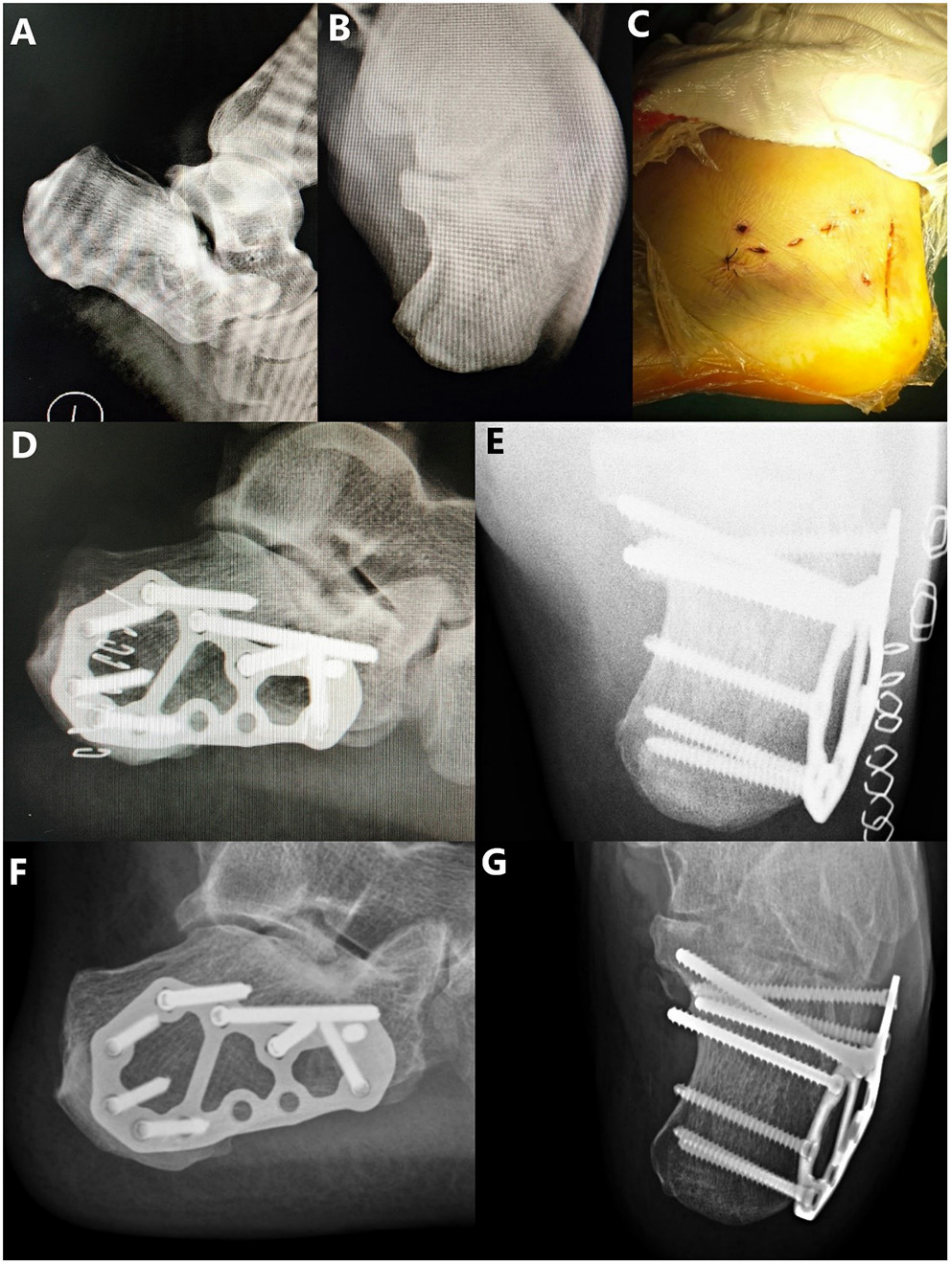
A 31-year-old male patient who fell from a height, resulting in a tongue-type intra-articular fracture of the calcaneum. **(A)** Preoperative lateral X-ray and **(B)** preoperative axial X-ray presenting an intra-articular tongue-type calcaneal fracture with subtalar articular surface collapse, loss of Böhler's angle and Gissane's angle, significantly decreased calcaneal height/length, and significantly increased calcaneal width. **(C)** Postoperative incision. **(D)** Postoperative lateral X-ray showing the reduction of the calcaneal length and height, Böhler's angle, and Gissane's angle. **(E)** Postoperative axial X-ray showing the reduction of the calcaneal width. **(F)** Lateral X-ray 12 months after the surgery. **(G)** Axial X-ray 12 months after the surgery.

**Figure 4:**
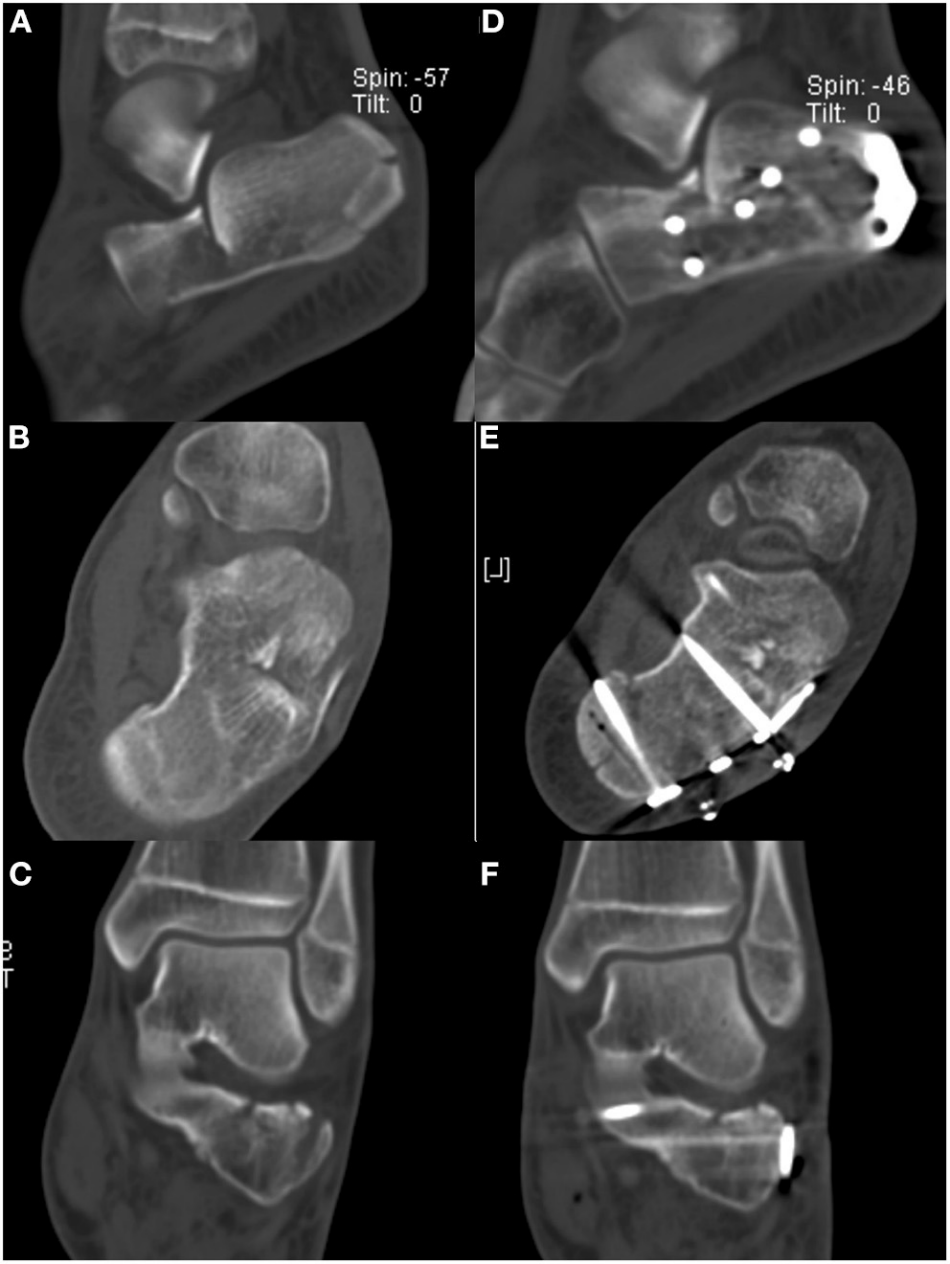
**(A–C)** The preoperative computed tomography (CT) scan of the foot from Figure 3 shows a Sanders type II fracture. **(D–F)** Postoperative CT scan shows the reduction of the articular surface and the restoration of the calcaneal width were satisfactory.

In the MILA group, closed reduction and provisional fixation with two Kirschner wires were achieved in the same way as aforementioned, after which a plate of a suitable size and position was placed on the lateral skin surface of the hindfoot. Kirschner wires (2.0 mm) were introduced into the calcaneus through these holes. A 4-cm longitudinal incision was made 1 cm in front of the calcaneal tubercle. The proper plate was placed with the periosteal elevator and fixed with screws percutaneously or through the incision using the C-arm. Finally, the incisions were closed (Figures 3, 4).

### Postoperative Management and Follow-Up

Radiographs and CT scans of the injured calcaneus were obtained after the surgical operation to evaluate the reduction and fixation. The two groups were instructed to perform postoperative exercises following the same rehabilitation protocol. Active and passive exercises, including the extension and plantar flexion of the toes and ankle with non-weight-bearing, were encouraged as soon as the pain could be endured on postoperative day 1. All patients had their stitches removed 2 weeks later and were instructed to touch the toe. Partial weight-bearing under protection was permitted 1 month postoperatively, full weight-bearing under protection was allowed 2 months postoperatively, and full weight-bearing without protection was suggested 3 months postoperatively when bone healing was seen on the radiological image. All patients underwent follow-up with routine physical examination and plain radiography (lateral and axial views) at 1, 3, 6, and 12 months postoperatively, and a final follow-up at 27 months. The implants were removed if the patient had indications for implant removal, such as posterior heel pain refractory to medical treatment and deep infection.

The following parameters were collected from both groups: operation time (OT), preoperative time (POT, the time from initial injury to surgery), hospital stay time (HST, the time from surgery to discharge), and blood loss. The length, width, and height of the calcaneus and the Gissane's angle, Böhler's angle, and varus or valgus angle were measured on plain radiographs. A normal Gissane's angle is 100°-130°, and a normal Böhler's angle is 25°-40° (6). When the posterior articular surface collapses due to calcaneal fracture, the Gissane's angle becomes larger, and the Böhler's angle becomes smaller. The visual analog scale (VAS) and American Orthopaedic Foot and Ankle Society (AOFAS) hindfoot scores were obtained to evaluate the function of the injured hindfoot (10). Postoperative complications were also noted.

### Statistical Analysis

Statistical analyses were performed using the IBM SPSS Statistics version 25 for Windows (IBM Corp. Armonk, NY, USA). Continuous data are reported as mean ± standard error of the mean, unless otherwise noted. Continuous variables were compared using independent samples *t*-test when the variables were approximately normally distributed, and non-normal distributions were analyzed using the Mann–Whitney U test. The chi-squared test or Fisher's exact test (*n* < 40 or *t* < 1) was used to evaluate the categorical parameters between the two groups. Statistical significance was set at *p* ≤ 0.05.

## Results

A total of 102 adult patients with intra-articular tongue-type calcaneal fractures were treated at our university hospital between September 2016 and July 2020. Sixteen patients were excluded owing to severe medical ailments (11 patients) and those aged >60 years (five patients). A total of 86 patients were included in this study, 9 of whom were lost to follow-up because they did not finish the follow-up protocol or could not be contacted. Therefore, 77 patients with an average age of 34.8 (range, 19–58) years, including 61 men and 16 women, participated in the final study. The 77 patients were followed up for an average of 27.4 (range, 21–30) months. Of the 77 patients, 32 and 45 were treated with closed reduction and PCS fixation (PCS group) and closed reduction and plating using the MILA (MILA group), respectively.

The PCS group included 25 men and 7 women with a mean age of 36.3 (range, 19–55) years. The injured side of the foot included 17 left and 15 right feet. The injury etiologies included fall from a height (20 fractures), traffic accident (nine fractures), and other causes (three fractures). In the MILA group, 36 men and nine women, with a mean age of 33.8 (range, 20–58) years were included. The injured side of the foot included 21 left and 24 right feet. The injury etiologies included fall from a height (29 fractures), traffic accident (11 fractures), and other causes (five fractures) (Table 1). The Sanders classification of all intra-articular calcaneal fractures in this study was type II. No significant differences were found between the two groups in terms of demographics, injured foot side, or injury etiology. Blood loss was lower in the PCS group (13.8 mL) than that in the MILA group (18.2 mL, *p* = 0.044). The average OT was shorter in the PCS group than that in the MILA group (60.5 vs. 86.2 min, *p* < 0.001). The average POT was shorter in the PCS group (1.4 days) than that in the MILA group (2.5 days, *p* < 0.001). In the PCS group, all patients were discharged on the second day postoperatively, unless it was a holiday, and they could not go through the discharge process. However, the longest HST was 4 days in the MILA group. By contrast, the average HST was shorter in the PCS group (1.3 days) than that in the MILA group (2.1 days, *p* < 0.001) (Table 1).

**Table 1:** Comparison of the patient characteristics of the percutaneous cannulated screw (PCS) fixation group and the minimally invasive longitudinal approach (MILA) group.

**General information**	**PCS group**	**MILP group**	***P*-value**
	*N* = 32	*N* = 45	
Age (years)	36.3 ± 11.8	33.8 ± 11.7	0.658
**Sex**			0.842
Male	25	36	
Female	7	9	
Body mass index	22.7 ± 2.4	22.8 ± 3.0	0.232
**Injury mechanism**			0.922
Fall from a height	20	29	
Traffic accident	9	11	
Others	3	5	
**Side of injured**			0.576
Left	17	21	
Right	15	24	
POT (days)	1.4 ± 0.5	2.5 ± 1.6	<0.001^*^
HST (days)	1.3± 0.6	2.1 ± 0.9	<0.001^*^
OT (min)	60.5 ± 11.6	86.2 ± 13.0	<0.001^*^
Blood loss (ml)	13.8 ± 8.8	18.2 ± 10.5	0.044^*^
**Complications**			
Wound complications	0	2 (4.4%)	0.508
Superficial infection	0	2 (4.4%)	0.508
Sural nerve injury	0	3 (6.7%)	0.262
Lateral hindfoot pain	2 (6.3%)	4 (8.9%)	1.000
Total	2 (6.3%)	9 (20.0%)	0.110
Reoperation	1 (3.1%)	4 (8.9%)	0.395

*OT, operation time; POT, preoperation time; HST, hospital stay time*.

* *The difference were statistically significant (p ≤ 0.05)*.

In the PCS group, two complications were observed: two patients complained of persistent pain on the lateral side of the injured foot. In the MILA group, nine complications were noted: two superficial infections, three sural nerve injuries characterized by skin numbness at the incision site, and four patients with lateral hindfoot pain. One and four patients in the PCS and MILA groups, respectively, underwent implant removal due to pain. None of the patients developed deep infections, hematomas, or osteomyelitis. Until the final follow-up, there was no subtalar arthritis observed. However, no significant differences were found in the complication rates between the two groups (Table 1).

As shown in Table 2, no significant differences were observed in the calcaneal Böhler's angle, Gissane's angle, varus or valgus angle, length, and height between the PCS and MILA groups before and after the surgery and during the follow-up period (*p* > 0.05). However, the calcaneal width was smaller in the PCS group than that in the MILA group during the postoperative and follow-up periods (*p* < 0.05).

**Table 2:** Radiographic results for the percutaneous cannulated screw (PCS) fixation group and the minimally invasive longitudinal approach (MILA) group.

	**PCS group**	**MILP group**	***P*-value**
**Gissane's angle (** **°** **)**			
Preoperative	116.8 ± 13.6	120.0 ± 13.6	0.228
Postoperative	123.5 ± 4.4	124.2 ± 4.9	0.363
1 month	122.5 ± 4.1	123.1 ± 4.9	0.587
3 months	122.5 ± 4.4	122.9 ± 4.9	0.922
6 months	122.5 ± 4.2	122.7 ± 4.8	0.913
12 months	122.6 ± 4.4	122.4 ± 4.7	0.598
Final follow-up	122.8 ± 4.6	122.5 ± 4.8	0.375
**Böhler angle (** **°** **)**			
Preoperative	11.1 ± 5.3	12.6 ± 7.3	0.591
Postoperative	25.0 ± 3.2	25.3 ± 3.7	0.897
1 month	24.8 ± 3.0	25.1 ± 3.6	0.804
3 months	24.8 ± 2.7	25.0 ± 3.4	0.865
6 months	24.9 ± 2.7	25.1 ± 3.4	0.885
12 months	24.7 ± 2.5	25.0 ± 3.2	0.864
Final follow-up	24.9 ± 2.7	25.1 ± 3.1	0.934
**Calcaneal length (mm)**			
Preoperative	82.7 ± 6.1	81.2 ± 5.3	0.562
Postoperative	84.2 ± 5.7	82.5 ± 6.2	0.438
1 month	82.5 ± 4.5	80.4 ± 5.9	0.148
3 months	82.9 ± 4.8	80.5 ± 5.8	0.096
6 months	83.7 ± 5.3	81.2 ± 6.1	0.064
12 months	83.4 ± 5.3	80.8 ± 5.6	0.099
Final follow-up	83.5 ± 5.6	80.9 ± 5.6	0.116
**Calcaneal width (mm)**			
Preoperative	45.4 ± 5.0	44.4 ± 3.1	0.251
Postoperative	43.8 ± 4.4	41.3 ± 3.2	0.015^*^
1 month	44.2 ± 4.7	41.3 ± 3.1	0.007^*^
3 months	43.7 ± 4.2	41.3 ± 4.5	0.022^*^
6 months	44.0 ± 3.9	41.2 ± 4.1	0.009^*^
12 months	43.9 ± 4.5	41.0 ± 4.0	0.010^*^
Final follow-up	43.7 ± 4.1	41.4 ± 4.1	0.042^3*^
**Calcaneal height (mm)**			
Preoperative	43.8 ± 5.0	42.1 ± 5.7	0.271
Postoperative	50.0 ± 4.1	48.9 ± 3.6	0.292
1 month	49.4 ± 4.5	48.0 ± 3.8	0.251
3 months	50.0 ± 4.1	48.4 ± 3.4	0.117
6 months	49.4 ± 3.8	48.2 ± 4.1	0.376
12 months	49.2 ± 4.7	48.1 ± 4.6	0.535
Final follow-up	49.5 ± 5.6	48.2 ± 4.0	0.379
**Varus (+) or valgus (–) (** **°** **)**			
Preoperative	3.5 ± 2.9	4.5 ± 3.1	0.108
Postoperative	2.1 ± 2.3	2.2 ± 1.6	0.113
1 month	2.4 ±2.3	2.5 ± 1.6	0.185
3 months	2.7 ± 1.6	2.6 ± 1.6	0.295
6 months	2.5 ± 1.8	2.7 ± 1.8	0.289
12 months	2.5 ± 2.2	2.8 ± 1.7	0.224
Final follow-up	2.6 ± 2.2	2.8 ± 1.7	0.275

* *The difference were statistically significant (p ≤ 0.05)*.

The functional outcomes are shown in Table 3. The VAS scores showed a decreasing trend, whereas the AOFAS hindfoot scores showed an increasing trend in both groups. The postoperative VAS scores in the PCS group were lower than those in the MILA group (*p* < 0.05). No significant differences were noted in the AOFAS hindfoot scores between the PCS and MILA groups at 1, 3, 6, and 12 months of follow-up and at the final follow-up (*p* > 0.05).

**Table 3:** Outcome scores for the percutaneous cannulated screw (PCS) fixation group and the minimally invasive longitudinal approach (MILA) group.

	**PCS group**	**MILP group**	***P*-Value**
**VAS**			
Preoperative	6.4 ± 1.2	6.6 ± 1.1	0.650
Postoperative	4.9 ± 0.6	5.5 ± 0.9	0.005^*^
1 month	3.3 ± 1.0	3.6 ± 0.9	0.155
3 months	0.6 ± 0.7	0.9 ± 0.9	0.201
6 months	0.3 ± 0.6	0.5 ± 0.6	0.149
12 months	0.2 ± 0.6	0.4 ± 0.6	0.149
Final follow-up	0.2 ± 0.5	0.3 ± 0.6	0.453
**AOFAS**			
1 month	63.6 ± 6.9	61.6 ± 7.7	0.280
3 months	75.3 ± 7.5	73.7 ± 7.2	0.824
6 months	85.5 ± 8.3	84.2 ± 9.0	0.544
12 months	88.9 ± 5.8	88.6 ± 5.7	0.464
Final follow-up	90.3 ± 6.4	88.2 ± 6.8	0.107

* *The difference were statistically significant (p ≤ 0.05)*.

## Discussion

Calcaneal fractures are the most common type of tarsal bone fractures; therefore, the treatment of calcaneal fractures has frequently been a hot topic of discussion among orthopedic surgeons. Several studies (11) have shown that the surgical treatment group has better functional outcomes than the non-surgical treatment group. The standard surgical approach is open reduction and internal fixation via an extensile L-shaped incision, which provides good results in anatomically reducing the subtalar joint (12). However, several postoperative complications present as challenges to orthopedic surgeons, such as deep wound infection, necrosis, and hematoma, owing to the large surgical incision (12). For this purpose, a number of minimally invasive techniques with less trauma to the soft tissues have been proposed (1, 3, 5, 6, 8–10). Whichever technique is applied, the principles of anatomic reduction and rigid fixation remain the same (13).

In our study, the first step in both fixation methods was closed reduction with Steinmann pins. Displaced intra-articular calcaneal fractures were divided by Essex–Lopresti (2) into tongue and joint depression types. Tongue-type fractures that could result in superior and posterior displacement of the displaced fragment are typically treated with percutaneous pin fixation because the fracture mass is large and can be easily reduced by leveraging technology (1, 7, 14). It is a critical step to restore the three-dimensional reconstruction of the calcaneal anatomy and the contact surface between the talus and calcaneus as much as possible for the surgery (3). Abdelgaid (15) confirmed that the function of the calcaneus and subtalar joint could be restored by percutaneous reduction and fixation in patients with displaced tongue-type intra-articular calcaneal fractures.

The radiographic parameters in the two different fixation approach groups were improved compared with the preoperative measurements. No statistically significant differences in radiographic parameters of the calcaneus, except for the calcaneal width, were found postoperatively or during the follow-up. Therefore, the role of PCS fixation in restoring the calcaneal width might be weaker than that of the MILA in our study; however, the two approaches have similar effectiveness in maintaining the reduction. Several studies have shown that a method that combines percutaneous reduction with cannulated screw fixation, which is applicable to all types of intra-articular calcaneal fractures, could achieve anatomic reduction and effectively prevent the loss of reduction (1, 15, 16). Studies have reported that screw fixation of calcaneal fractures has biomechanical stability similar to that of the plating technique (17, 18). However, in the studies by Kir et al. (3) and Feng et al. (10), compared with cannulated screw fixation, the plate fixation procedure had its own advantages in improving the calcaneal width. Kir et al. believed that this may be partially explained by the compressive force and better maintenance of the stabilization of the plate compared with that by the screw. The results of our study are consistent with those of Kir et al. and Feng et al. in that calcaneal widening could be improved by plate fixation. Furthermore, the restoration of the calcaneal varus was dissatisfactory in two patients in the PCS group and four patients in the MILA group. None of the six patients, however, experienced long-term postoperative pain.

With respect to short-term clinical outcomes, PCS fixation was superior to MILA. As a type of longitudinal fracture that exits the calcaneal tuberosity posteriorly and involves a portion of the articular surface, tongue-type calcaneal fractures are often superiorly and dorsally displaced by the pull of the gastroc-soleus complex (19). Early surgery is necessary because displacement may place significant strain on the relatively thin skin of the heel, eventually leading to necrosis (20). Minimally invasive plate fixation could not be performed until the hindfoot swelling subsided because untimely surgery increased the risk of incisional complications, for which a shorter POT was a strong piece of evidence. The shorter HST and lower postoperative VAS scores indicated that PCS fixation sped up the postoperative rehabilitation of patients and created favorable conditions for postoperative functional exercise.

All the patients in our study were encouraged to begin early postoperative rehabilitation following the same rehabilitation protocol and received regular follow-up. In the long-term follow-up, no statistically significant differences were noted in the VAS and AOFAS hindfoot scores between the two groups, indicating that the curative effects of the two methods were similar. This is consistent with the results of several previously reported studies (21, 22).

In our study, postoperative complications and the probability of returning to the operating room were not significantly different between the PCS and MILA groups. However, PCS fixation was associated with a low risk of wound-healing complications. The shorter OT and less interference with the blood supply to the lateral aspect of the hindfoot in the PCS group may help reduce the incidence of infection. Sural nerve injury was more likely to occur in the MILA group owing to the position of the nerve. Furthermore, a higher proportion of patients with the MILA complained of lateral hindfoot pain than those with PCS fixation. Clare and Crawford (23) reported that peroneal tenosynovitis and stenosis, which may cause lateral hindfoot pain, mainly result from the expanded lateral wall of the calcaneus. Consequently, the peroneal tendons subluxate and cause them to impinge on the tip of the fibula or cause frank dislocation over the fibula (10, 23). However, peroneal tenosynovitis and stenosis were more likely to occur in the MILA group with a smaller mean calcaneal width after surgery than that in the PCS group in this study. In the MILA group, four patients reported that the pain had lessened after the implants were removed. Related studies have suggested that soft tissue adhesions and implant prominence can contribute to similar symptoms following surgical management (23, 24). Furthermore, Chotikkakamthorn et al. (25) believed that peroneal tendon irritation to the plate was the primary source of pain because screw fixation was unlikely to impinge the peroneal tendon. Thus, we postulate that pain could be caused by a combination of these two factors. However, this hypothesis still needs to be tested in future studies.

This study had a few limitations. First, this was a single-center retrospective cohort study with a small sample size. Therefore, multicenter randomized controlled trials are required to avoid selection bias. Second, owing to the relatively short follow-up time, long-term complications, such as subtalar arthritis, could not be assessed. Finally, errors in the patient records during follow-up may have led to trial errors.

## Conclusion

Plate fixation was found to be better than screw fixation in the recovery of the calcaneal width after closed reduction for the treatment of tongue-type calcaneal fractures. However, PCS fixation had a shorter OT, less blood loss, shorter time between initial injury and surgery, shorter time between surgery and discharge, and lower postoperative VAS scores than the MILA. Under the same rehabilitation program, the two approaches showed similar effectiveness in maintaining reduction.

## Data Availability Statement

The raw data supporting the conclusions of this article will be made available by the authors, without undue reservation.

## Ethics Statement

The studies involving human participants were reviewed and approved by the Medical Ethics Committee of the Peking University Third Hospital. The patients/participants provided their written informed consent to participate in this study. Written informed consent was obtained from the individual(s) for the publication of any potentially identifiable images or data included in this article.

## Author Contributions

YL, FZ, YT, and YG conceived and designed the study. YC, SG, and YZ collected the clinical data. YC and ZC performed the statistical analysis. YC and XX drafted the manuscript. YL revised the paper. All authors contributed to the manuscript and approved the submitted version.

## Funding

This work was supported by the National Key R&D Program of China (No. 2018YFF0301102).

## Conflict of Interest

The authors declare that the research was conducted in the absence of any commercial or financial relationships that could be construed as a potential conflict of interest.

## Publisher's Note

All claims expressed in this article are solely those of the authors and do not necessarily represent those of their affiliated organizations, or those of the publisher, the editors and the reviewers. Any product that may be evaluated in this article, or claim that may be made by its manufacturer, is not guaranteed or endorsed by the publisher.
